# A Versatile and Robust Platform for the Scalable Manufacture of Biomimetic Nanovaccines

**DOI:** 10.1002/advs.202002020

**Published:** 2021-05-01

**Authors:** Hanze Hu, Chao Yang, Fan Zhang, Mingqiang Li, Zhaoxu Tu, Lizhong Mu, Jianati Dawulieti, Yeh‐Hsing Lao, Zixuan Xiao, Huize Yan, Wen Sun, Dan Shao, Kam W. Leong

**Affiliations:** ^1^ Department of Biomedical Engineering Columbia University New York NY 10027 USA; ^2^ Institutes for Life Sciences School of Biomedical Sciences and Engineering South China University of Technology, Guangzhou International Campus Guangzhou Guangdong 510630 China; ^3^ National Engineering Research Center for Tissue Restoration and Reconstruction Key Laboratory of Biomedical Engineering of Guangdong Province South China University of Technology Guangzhou 510006 China; ^4^ Laboratory of Biomaterials and Translational Medicine The Third Affiliated Hospital Sun Yat‐sen University Guangzhou Guangdong 510006 China; ^5^ School of Energy and Power Engineering Dalian University of Technology Dalian Liaoning 116024 China; ^6^ State Key Laboratory of Fine Chemicals Dalian University of Technology Dalian Liaoning 116024 China; ^7^ Department of Systems Biology Columbia University New York NY 10032 USA

**Keywords:** biomimetics, cancer vaccine, cell membrane, flash nanocomplexation, mesoporous silica nanoparticles

## Abstract

Biomimetic strategies are useful for designing potent vaccines. Decorating a nanoparticulate adjuvant with cell membrane fragments as the antigen‐presenting source exemplifies, such as a promising strategy. For translation, a standardizable, consistent, and scalable approach for coating nanoadjuvant with the cell membrane is important. Here a turbulent mixing and self‐assembly method called flash nanocomplexation (FNC) for producing cell membrane‐coated nanovaccines in a scalable manner is demonstrated. The broad applicability of this FNC technique compared with bulk‐sonication by using ten different core materials and multiple cell membrane types is shown. FNC‐produced biomimetic nanoparticles have promising colloidal stability and narrow particle polydispersity, indicating an equal or more homogeneous coating compared to the bulk‐sonication method. The potency of a nanovaccine comprised of B16‐F10 cancer cell membrane decorating mesoporous silica nanoparticles loaded with the adjuvant CpG is then demonstrated. The FNC‐fabricated nanovaccines when combined with anti‐CTLA‐4 show potency in lymph node targeting, DC antigen presentation, and T cell immune activation, leading to prophylactic and therapeutic efficacy in a melanoma mouse model. This study advances the design of a biomimetic nanovaccine enabled by a robust and versatile nanomanufacturing technique.

## Graphical abstract

Flash nanocomplexation (FNC) is exploited to produce cell membrane‐biomimetic nanotherapeutics in a robust, versatile, reproducible, and scalable manner. Cancer cell membrane‐coated, CpG‐loaded mesoporous silica nanoparticles fabricated using FNC exhibit better lymph node targeting, T‐cell activation, anticancer immunoresponse, and combined immunotherapeutic outcome than sonication‐produced counterparts.

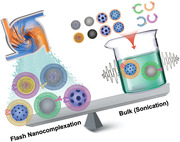

Biomimetic strategies are critical to developing sophisticated nanoparticle‐based therapeutics that can negotiate biological barriers.^[^ ^1^ ^]^ Cell membrane coating technology offers a promising solution to the challenges of therapeutic delivery,^[^ ^1a–d^ ^]^ integrating the biological features of cell membranes with the functional versatility of nanomaterials.^[^ ^1e^ ^]^ Production involves coating synthetic nanoparticle backbone materials with a naturally‐derived cell membrane layer to form a biomimicking ensemble.^[^ ^2^ ^]^ These nanotherapeutics have shown advantageous physical properties, such as improved stability and longer circulation times, and intrinsic functionalities inherited from the donor cell source such as toxin neutralization, homologous targeting, and immune invasion.^[^ ^3^ ^]^ However, producing regulatory agency‐approved cell membrane‐coated nanomaterials requires an additional level of manufacturing sophistication. Current approaches to fabricating cell membrane‐coated nanomaterials rely on two main strategies: extrusion and sonication.^[^ ^4^ ^]^ Extrusion produces homogeneous coatings and uniform size, but it is prohibitively time‐consuming; sonication offers a facile approach to produce sufficient product, but the quality control can be compromised in several ways, and the coating outcomes may vary on different protocols.^[^ ^2^ ^]^ The difficulty of fabricating cell membrane‐coated nanomaterials in a facile, large‐scale, and reproducible manner restricts their prospects for clinical and industrial translation. Standardized formulation techniques are needed to ensure that these biomimetic materials can be produced in a timely manner with minimal batch‐to‐batch variation and using good manufacturing practice. Fundamentally, the challenge is to develop an efficient and reliable cell membrane coating process.

To surmount these obstacles, we took advantage of a recently developed technology termed flash nanocomplexation (FNC).^[^ ^5^ ^]^ FNC involves turbulent mixing and self‐assembly of nanomaterial ingredients in defined microchambers. It can be applied to the manufacture of a variety of nanoformulations for therapeutic and bioimaging applications,^[^ ^6^ ^]^ and provides a robust platform that may expedite the translation of nanotherapeutics. FNC exploits the dynamic mixing of nanocomposites that undergo self‐assembly via physical forces, such as electrostatic interactions,^[^ ^7^ ^]^ in which case charged nanomaterials assemble to form nanoparticles (NPs) or to modify a NP surface.^[^ ^8^ ^]^ Uniform lipid‐coated nanoparticles have been fabricated using FNC by optimizing the lipid/NP mixing ratio, flow rate, and lipid composition.^[^ ^8^, ^9^ ^]^ We hypothesized that FNC could be applied to the mixing of cell membrane fragments and synthetic backbone materials for the robust and scalable production of cell membrane‐coated NPs. In addition, by using multi‐inlet vortex mixers (MIVM) in the mixing microchamber, the kinetic energy of multiple inlet jets can be applied to transport cell membrane fragments and synthetic backbone materials into regions of small turbulent eddies and intershearing layers for better flow convection and hence faster coating.^[^ ^10^ ^]^ The mixing ratio and flow rate can then be tuned to produce a uniform coating.

Herein, we demonstrate the use of FNC for coating a variety of core nanomaterials with cell membranes, and validate a FNC‐produced cancer vaccine, B16‐F10 cancer cell membrane‐coated mesoporous silica nanoparticles (MSNs) loaded with the adjuvant cytosine‐phosphate‐guanosine (CpG), in the in vitro and in vivo models. This study is a proof‐of‐concept for using the FNC‐based protocol for efficient, scalable, and reproducible preparation of cell membrane‐coated NPs.

The first step was to characterize and optimize the FNC cell membrane‐coating process. We systematically compared coating outcomes through both FNC and sonication approaches using different core materials and various cell membrane types (**Figure** **1**a). Ten particulate cores with different size, pore structure, and surface charge were prepared, including poly(lactic*‐co‐*glycolic acid) (PLGA), polyethyleneimine (PEI)‐plasmid, and silica particles (Table S1, Supporting Information). Different cell types (cancer, nonimmune, and immune cells) were exploited to obtain the cell membrane fragments. The core–shell structure of the resulting cell membrane‐coated particles was confirmed using electron microscopy (Figures S1 and S2, Supporting Information). Increases in particle size after coating and changes in particle surface charge were observed through dynamic light scattering (Figures S3 and S4, Supporting Information). Figure 1b shows a plot of size change of various bare particles (Figure S5, Supporting Information) immediately after coating versus change at 2 weeks after coating in phosphate‐buffered saline solution; it indicates differences in membrane coating homogeneity and particle stability for between the products of the two methods. Across a spectrum of NPs of cell membranes, FNC products showed a smaller size change and better particle colloidal stability than the bulk‐sonication products among many particle types. Specifically, for some of the MSN subtypes and PLGA nanoparticles, the aggregation seen at day 14 was reduced. The lower polydispersity index (PDI) for FNC products also suggests good particle stability. In the sonication method, ultrasound wave‐energy pulverizes the cell membrane structure, and membrane fragments reassemble around the nanoparticle backbone.^[^ ^11^ ^]^ Although easy to operate, the coating protocol is not standardized for bulk containers because the sonication power‐frequency may not be uniform or optimized, and the quantity of cell membrane charge over the backbone surface may not be well‐controlled.^[^ ^2^, ^12^ ^]^ While electrostatic interactions are the driving force in both approaches, the FNC method achieves fast and homogeneous coating by using dynamic mixing,^[^ ^8b,c^ ^]^ including turbulent intershearing flow in the microchamber. This dynamic mixing can break cell membranes into small fragments and interweave the components to achieve even coating.

**Figure 1:**
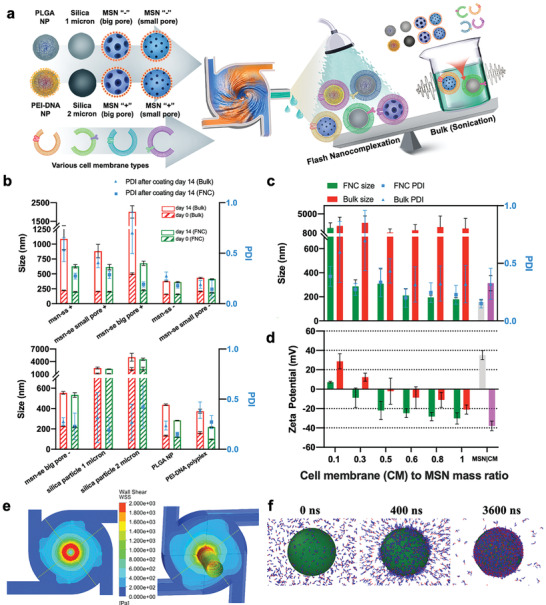
Fabrication of cell membrane‐coated nanoparticles using flash nanocomplexation. a) Schematic illustration of FNC cell membrane coating. b) Comparison of FNC and bulk sonication methods on PDI and stability of membrane‐coated nanoparticles. Characterization of membrane‐coated MSNs using different membrane‐to‐MSN ratios in terms of c) size and PDI, and d) Zeta potential. e) Analysis of shear stress within the MIVM (Re > 2000). f) MD simulation of homogeneously distributed anionic lipids interact with the cationic silica NP.

Cell membrane coating process relies on electrostatic interactions between cell membrane fragments and core materials to form “right‐side‐out” membrane‐coated products.^[^ ^13^ ^]^ Previously, it has been difficult to apply cell membranes uniformly to cationic surfaces due to the collapse of the fluidic lipid bilayer and disordered structure, resulting in particle aggregation.^[^ ^12^ ^]^ We assumed that the mixing time (*τ*_mixing_), within which cell membrane fragments and backbone materials are mixed homogenously, is much longer than the interacting time (*τ*_coating_) in the bulk mixing case. Thus, only a fraction of the cell membrane fragments are available to participate in coating the highly positive‐charged backbones, leading to heterogeneous coating and irreversible aggregation. Hypothesizing that FNC can improve the uniformity of coating by reducing *τ*_mixing_ as reported recently,^[^ ^14a^ ^]^ we compared the FNC and bulk sonication methods in coating cationic MSNs. Importantly, we invited operators who are new to the protocol of performing cell membrane coating on cationic MSNs using both coating methods. FNC products showed good dispersion (lower PDI) at many membrane/MSN ratios (Figure 1c), while some sonication products showed significantly higher PDI values. FNC also yielded better nanoparticle charge conversion than sonication in majority of the membrane to MSN ratio groups, which suggests a more complete cell‐membrane coating (Figure 1d). The surface charge of a completely coated nanoparticle resembles the intrinsic charge of cell‐membrane vesicles, whereas incomplete coating partially reveals the charge of nanoparticles and neutralizes the zeta potential. These results indicate that cationic nanoparticles can also be coated with cell membranes through flash‐based technique.

To investigate the coating process involved in the turbulent micromixing chamber that is difficult to capture experimentally, we performed computational fluidic dynamics to characterize flow profile in the MIVM (Figure S6, Supporting Information), and utilized molecular dynamics (MD) simulation to study molecular interaction of lipid fragments and cationic MSN. We found the shear stress generated within the MIVM has the magnitude of greater than 1 kPa (Figure 1e), which is sufficient to pulverize cell‐membrane micelles into fragments.^[^ ^14b^ ^]^ Subsequently, we performed MD based on scattered anionic lipid fragments and a silica NP with a positively charged surface that resembles our synthesized amine‐modified MSNs (MSN_NH2_) in a cubic simulation‐box (Figure 1f). Within milliseconds, evenly distributed lipids diffuse and cover the MSN surface homogeneously owing to electrostatic and hydrophobic interaction.

Efficient manufacturing is crucial to clinical translation of biomimetic therapeutics.^[^ ^15^ ^]^ We investigated the scale‐up capability of the FNC procedure by testing the rate of production of cell membrane‐coated MSNs. Using a four‐inlet MIVM with the total flow rate of 166 mL min^−1^, 120 g of biomimetic nanoproducts can be prepared daily (Figure S7, Supporting Information). Note the rate of production in a laboratory setting is much lower when using extrusion or sonication. Membrane‐coating efficacy was also compared between FNC and sonication method (Table S2, Supporting Information). While there is no accurately reported number regarding cell membrane‐coating yield by both sonication or extrusion method, the extrusion technique generates ≈5 mg of coated nanoproduct per batch in a general laboratory setting. Cell membrane‐coating achieved through the bath sonication is able to coat up to 50 mg of nanoparticles. However, further scale‐up of production using this method is limited. The coating efficacy and outcome are hinged upon nanoparticle solution to membrane solution volume ratio, volume size, sonication time, and differences in sonication power. Different operators may handle the coating procedure differently, which could lead to a variety of production outcomes. Of note, high sonication power may also affect the configuration and structural integrity of some nanoformulations, which may limit the application of the sonication‐based method on some biomimetic products preparation.^[^ ^15d^ ^]^

Cell membrane‐coated nanoformulations show promise for use in cancer immunotherapy.^[^ ^16^ ^]^ Cancer vaccines can be created by combining tumor‐associated antigens and immune‐activating adjuvants.^[^ ^17^ ^]^ The presentation of tumor‐associated antigens on cancer cell membrane‐coated backbone materials together with the delivery of adjuvants, such as CpG, could achieve lymph node targeting and generate tumor‐specific immune responses.^[^ ^16^, ^18^ ^]^ Although there is a booming interest in applying biomimetic nanotechnology to cancer immunotherapy, the manufacture of nanoformulations faces persistent challenges in large‐scale, reproducible, and efficient production.^[^ ^1d^ ^]^ We previously fabricated multiple stimuli‐responsive and biodegradable diselenide‐bridged MSNs for efficient delivery of biomacromolecules for cancer therapy.^[^ ^19^ ^]^ Here, for the first time, we integrate one of these big‐pore MSNs as the carrier of the adjuvant CpG, and coat them with cancer cell membrane containing tumor‐specific antigens to form a biomimetic nanovaccine (MSN‐CpG@CM) (**Figure** **2**a). CpG 1826 was encapsulated in MSN_NH2_ for maximum loading. B16‐F10 mouse melanoma cell was selected as the cell membrane source. We compared the FNC with bulk mixing/sonication approaches on biomimetic cancer vaccine production and systematically evaluate their therapeutic efficacy in vitro and in vivo.

**Figure 2:**
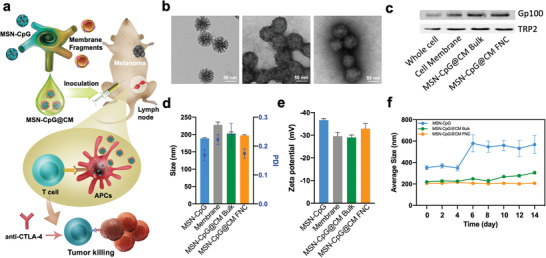
Fabrication and characterization of cancer cell membrane‐coated cancer vaccine. a) Schematic illustration of B16‐F10 cancer cell membrane‐coated, CpG‐loaded MSNs (MSN‐CpG@CM) produced by FNC. b) TEM images of MSN‐CpG, bulk MSN‐CpG@CM, and FNC MSN‐CpG@CM. c) Gp100 and TRP2 expressions on MSN‐CpG@CM. d) Size and PDI. e) Zeta potential. f) Long‐term stability of MSN‐CpG@CM. Data represent mean ± SD (*n* = 3) for panels d–f).

We coated CpG‐loaded MSNs with B16‐F10 cell membrane fragments using the FNC platform with a turbulent MIVM or using bulk sonication. A membrane‐to‐NP mass ratio of 1:1 was selected since this value has been evaluated and often reported for cell membrane coating.^[^ ^20^ ^]^ The surface morphologies of the CpG‐loaded MSNs before (MSN‐CpG) and after coating (MSN‐CpG@CM) using the two methods are shown in transmission electron microscopy (TEM) images (Figure 2b). The tumor‐associated antigens are specific for melanoma targeting, and the presence of antigens gp100 and TRP2 in the membrane coating of the MSN‐CpG@CM particles was confirmed by Western blot (Figure 2c). Other B16‐F10 cell membrane proteins were also found in the coating of the MSN‐CpG@CM particles (Figure S8, Supporting Information). The sodium dodecyl sulphate‐polyacrylamide gel electrophoresis (SDS‐PAGE) protein analysis and the Western Blot evaluation for both gp100 and TRP2 protein expression also suggested that the proteins could be well preserved after the FNC membrane coating. An increase in nanoparticle size and changes in zeta potential after coating also indicated the presence of a cell membrane coating (Figure 2d,e). Smaller PDI values were observed for the MSN‐CpG@CM particles when using FNC than when using bulk sonication. For both methods, aggregation was observed for naked NPs over the 2‐week stability evaluation period, whereas the membrane‐coated NPs maintained consistent size (Figure 2f). The improved colloidal stability might be explained by the symmetrical charge repulsion between cell membrane‐coated NPs. In addition, we observed a high CpG cargo‐loading capacity and GSH/reactive oxygen species dual‐responsive CpG release for the MSN‐CpG@CM (Figure S9, Supporting Information), indicating their potential for use in stimuli‐responsive immunotherapeutic delivery.

Safety is crucial for developing a biomimetic nanovaccine. We confirmed that MSN‐CpG@CM at <50 µg mL^−1^ showed no significant cytotoxicity using two types of antigen‐presenting cells (APCs) (Figure S10, Supporting Information). A high degree of intracellular colocalization of CpG‐loaded MSNs and cancer cell membrane proteins were observed in endosomes/lysosomes after 3 h of uptake (**Figure** **3**a), further verifying the structural integrity and stability of the MSN‐CpG@CM. We then compared the uptake of MSN‐CpG@CM by bone marrow‐derived dendritic cells (BMDCs) (Figure 3b). Both MSN‐CpG@CM groups (prepared using either FNC or bulk sonication) showed improved CpG uptake by DC cells versus naked MSN‐CpG, demonstrating the APC‐targeting effect in vitro. MSN‐CpG@CM were then injected into mice via the foot pad, and nanovaccines were observed in the popliteal lymph node after 1 h of administration. The fluorescence signal from dye‐labeled CpG peaked at 12 h after injection, and started to decrease at 24 h (Figure 3c). Quantification of mean fluorescence intensity of free CpG, naked MSN‐CpG, and MSN‐CpG@CM in the lymph node confirmed this observation (Figure 3d). Greater lymph node accumulation of MSN‐CpG@CM was observed when using FNC method than when using sonication, further confirmed through dye‐labeled membranes (Figure S11, Supporting Information). In terms of targeting APC internalization, both DCs and macrophages preferred endocytosing MSN‐CpG@CM to MSN‐CpG, indicating specific recognition of tumor antigens by the APCs. Greater CpG accumulation in DCs and macrophages from the popliteal lymph node was observed when using FNC than when using sonication (Figure 3e). Collectively, these results indicated that FNC produced a cell membrane‐coated cancer vaccine with better lymph node targeting and APC accumulation than sonication.

**Figure 3:**
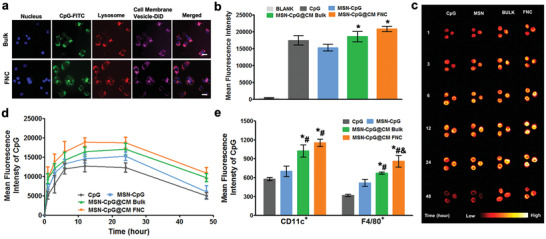
Antigen‐presenting cell uptake and lymph node targeting of cell membrane‐coated CpG‐loaded MSNs (MSN‐CpG@CM). a) Intracellular colocalization of DiD‐labeled B16‐F10 membrane modifications and FITC‐labeled CpG‐loaded MSNs in bone marrow‐derived dendritic cells (BMDCs) after incubation for 3 h. Scale bars, 10 µm. b) Relative fluorescence intensity of BMDCs after incubation with MSN‐CpG@CM for 3 h. Data represent mean ± SD (*n* = 3, *p < 0.05 vs MSN‐CpG group). c) Fluorescence imaging of popliteal lymph node at indicated time points after footpad injection of free CpG, naked MSN‐CpG, or MSN‐CpG@CM produced using bulk sonication or FNC methods. d) Quantitation of fluorescence intensity from Cy5.5‐labeled CpG in the popliteal lymph node. e) Uptake of Cy5.5‐labeled MSN‐CpG@CM by DCs and macrophages in the lymph node at 24 h after injection. Data represent mean ± SD (*n* = 3, *p < 0.05 vs CpG group, ^#^ *p *< 0.05 vs MSN‐CpG group, ^&^ *p *< 0.05 vs bulk MSN‐CpG@CM group).

As the DC maturation and the generation of antigen‐specific T cells are important for produced nanovaccine to activate the immune response, we assessed the MSN‐CpG@CM‐induced DC maturation by measuring the in vitro expression of the costimulatory markers CD80, CD40, and CD86 as well as the APCs secretion of TNF‐ɑ, IL‐6, and IL‐12 (Figure S12, Supporting Information). In lymph node, CpG alone and MSN‐CpG induced less potent DC maturation than MSN‐CpG@CM (**Figure** **4**a). [MSN‐CpG@CM]_FNC_ induced greater DC maturation and secretion of IL‐6 and IL‐12 than [MSN‐CpG@CM]_sonication_ (Figure 4a; and Figure S13, Supporting Information). Importantly, [MSN‐CpG@CM]_FNC_ promoted greater generation of T cells specific for gp100 than [MSN‐CpG@CM]_sonication_ (Figure 4b), indicating better presentation of gp100 antigen for T‐cell activation. Together, these results indicated that the FNC‐produced cancer vaccine could stimulate DC antigen presentation and the tumor antigen‐specific T cell response.

**Figure 4:**
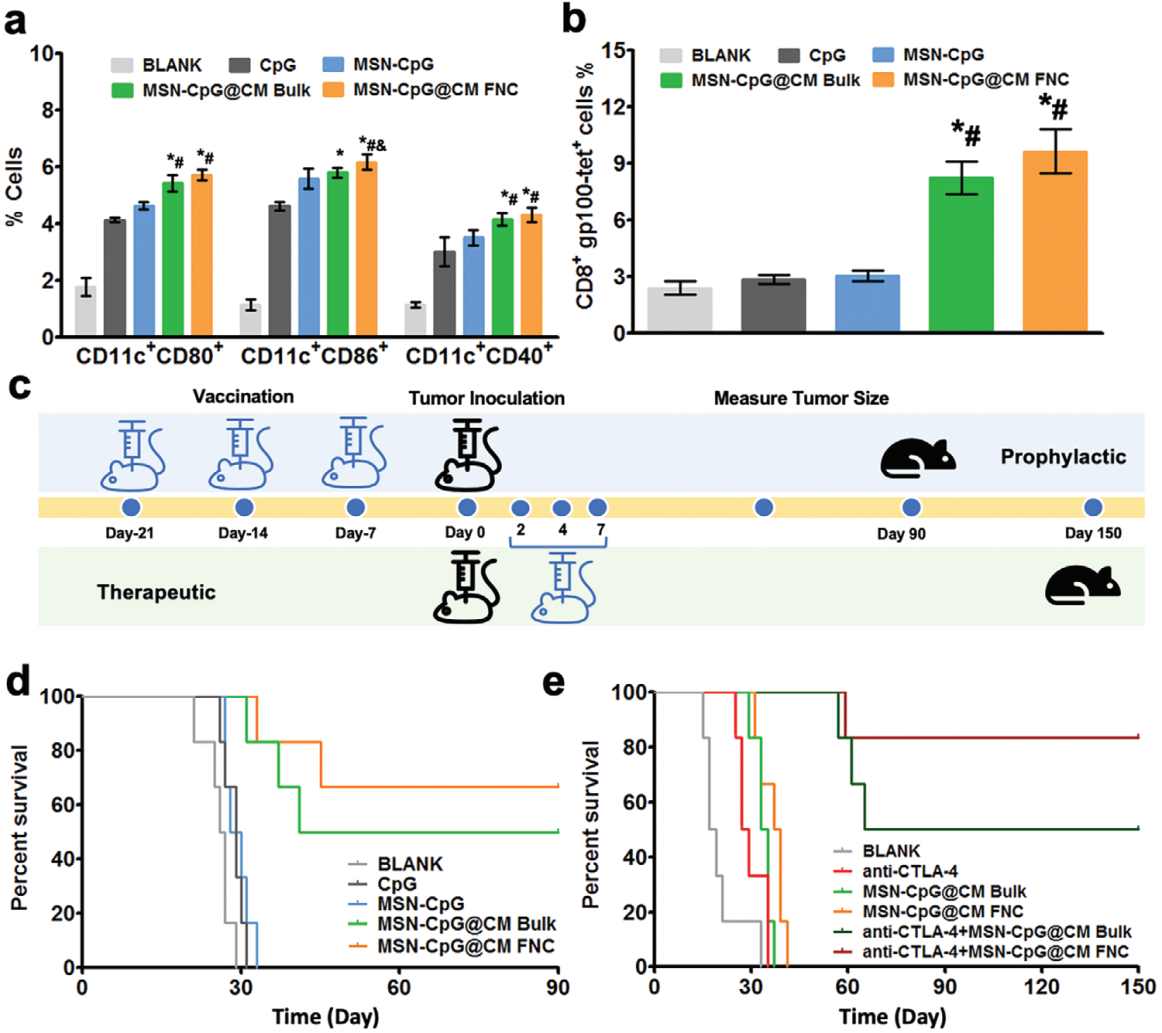
Anticancer immunoresponse in melanoma mouse model. a) Quantification of DC maturation markers CD40, CD80, and CD86 in the popliteal lymph node (*n* = 3). b) Tetramer staining analysis of gp100‐specific T cells (*n* = 3). c) Illustration of the prophylactic and therapeutic experiment design. d) Prophylactic effect of nanovaccines on survival rate (*n* = 6). e) Therapeutic effect of nanovaccines with or without the checkpoint blockade inhibitor anti‐CTLA‐4 on survival rate (*n* = 6). Data represent mean ± SD (*p < 0.05 vs CpG group, ^#^ *p *< 0.05 vs MSN‐CpG group, ^&^ *p *< 0.05 vs bulk MSN‐CpG@CM group).

Strong prophylactic and therapeutic effects against tumor reflect the potency of the nanovaccine. First, we evaluated APCs responses, specific immune activation, and prophylactic tumor growth inhibition in vivo using a wide‐type B16‐F10 murine model (Figure 4c,d). Mice were vaccinated using different nanoformulations and tumor growth was monitored for up to 40 days. MSN‐CpG and free CpG had no significant protective benefit, consistent with previous studies;^[^ ^16^, ^18^ ^]^ both treatments showed a median survival of 29 d, similar to the median 26.5 d survival for the negative control. Both FNC‐ and sonication‐produced MSN‐CpG@CM groups showed tumor growth inhibition, but the FNC‐produced vaccine had a better inhibitory effect and longer survival (Figure S14, Supporting Information). The improved antitumor response of our FNC‐based nanovaccines was attributed to the better lymph node targeting due to enhanced colloidal stability of the nanoparticles. Next, we assessed the therapeutic performance of the MSN‐CpG@CM with and without the immune checkpoint‐blocking antibody anti‐CTLA‐4. Without anti‐CTLA‐4, the median survival was extended from 18 d for the blank control group to 34 d for the [MSN‐CpG@CM]_sonication_ group and 38 d for the [MSN‐CpG@CM]_FNC_ group (Figure 4c,e). With anti‐CTLA‐4, the median survival was over 150 days for both FNC and sonication MSN‐CpG@CM groups, indicating that combined immunotherapy produced synergistic antitumor effects. Importantly, we evaluated the CD8+/CD4+ ratio, the percentage of cytotoxic T lymphocytes and the percentage of regulatory T cells within the tumors using different nanoformulations. Consistently, the combined therapy using FNC‐produced nanovaccines with anti‐CTLA‐4 led to the most potent antitumor effect among the groups, correlating with the increased CD8+/CD4+ ratio, a higher cytotoxic T lymphocyte number, and a reduced regulatory T cell number within the tumors (Figures S15 and S16, Supporting Information). For future studies, the infiltration of other immune cell types and the recruitment and repolarization of tumor‐associated macrophages in the tumor microenvironment would be worth investigating.

In summary, we have demonstrated a nanoformulation platform for fabricating diverse cell membrane‐based NPs in a facile, reproducible, and scalable manner. The FNC platform leverages dynamic turbulent mixing to homogeneously blend and uniformly distribute cell membrane fragments around NP surfaces. We establish that FNC can be used to coat both negatively‐ and positively‐charged particles with cell membranes, and it reduces batch‐to‐batch variation and production time compared with the conventional sonication‐coating process. The largely automated process should facilitate standardization. In addition to achieving a higher throughput, the FNC process may also improve the potency of the nanotherapeutics. FNC‐producing MSNs loaded with CpG adjuvant and coated with a cancer cell membrane exhibited enhanced accumulation in lymph nodes and immune activation, and greater tumor growth inhibition alone and in combination with the immune checkpoint‐blocking antibody anti‐CTLA‐4 in a melanoma model. This study addresses the challenge of manufacturing for cell membrane‐coated nanotherapeutics. For future studies, an in‐depth mechanistic investigation of the cell membrane‐coated NP in generating an immune response and in tissue biodistribution and intracellular trafficking would be worth exploring.

## Experimental Section

Materials and experimental details are provided in the Supporting Information.

All animals received care in compliance with the guidelines outlined in the Guide for the Care and Use of Laboratory Animals, and the procedures were approved by the South China University of Technology Animal Care and Use Committee.

## Conflict of Interest

The authors declare no conflict of interest.

## Supporting information

Supporting Information

## Data Availability

Research data are not shared.
