# Supplementary material for: A Versatile and Robust Platform for the Scalable Manufacture of Biomimetic Nanovaccines
Source: Adv Sci (Weinh). 2021 May 1;8(15):2002020. doi: 10.1002/advs.202002020 (PMC8336609; doi:10.1002/advs.202002020)
Supplement: Supplementary file 1 — Supporting Information [file ADVS-8-2002020-s001.pdf]

© 2021 Wiley-VCH GmbH

ADVANCED SCIENCE
Open Access

Supporting Information

for *Adv. Sci.,* DOI: 10.1002/advs.202002020

A Versatile and Robust Platform for the Scalable Manufacture of Biomimetic Nanovaccines

Hanze Hu, Chao Yang, Fan Zhang, Mingqiang Li, Zhaoxu Tu, Lizhong Mu, Jianati Dawulieti, Yeh-Hsing Lao, Zixuan Xiao, Huize Yan, Wen Sun, Dan Shao, and Kam W. Leong**

Supporting Information

A Versatile and Robust Platform for the Scalable Manufacture of Biomimetic Nanovaccines

Hanze Hu, Chao Yang, Fan Zhang, Mingqiang Li, Zhaoxu Tu, Lizhong Mu, Jianati Dawulieti, Yeh-Hsing Lao, Zixuan Xiao, Huize Yan, Wen Sun, Dan Shao, and Kam W. Leong**

H. Hu, Dr. C. Yang, Dr. Y.-H. Lao, Dr. Z. Tu, Z Xiao, H. Yan, Prof. K. W. Leong
Department of Biomedical Engineering
Columbia University
New York, NY 10027, USA
E-mail: kam.leong@columbia.edu

Dr. C. Yang, F. Zhang, Dr. J. Dawulieti, Dr. Z. Tu, Prof. D. Shao
Institutes for Life Sciences, School of Biomedical Sciences and Engineering, South China University of Technology Guangzhou International Campus
Guangzhou, Guangdong 510630, China
E-mail: stanauagate@outlook.com

Prof. M. Li
Laboratory of Biomaterials and Translational Medicine
The Third Affiliated Hospital
Sun Yat-sen University
Guangzhou, Guangdong 510006, China

Prof. L. Mu
School of Energy and Power Engineering
Dalian University of Technology
Liaoning, Dalian 116024, China

Prof. W. Sun
State Key Laboratory of Fine Chemicals
Dalian University of Technology
Liaoning, Dalian 116024, China

Dr. C. Yang, Dr. J. Dawulieti, Prof. D. Shao
National Engineering Research Center for Tissue Restoration and Reconstruction, Key Laboratory of Biomedical Engineering of Guangdong Province,
South China University of Technology,
Guangzhou 510006, China

Prof. K. W. Leong
Department of Systems Biology
Columbia University
New York, NY 10032, USA

H. H. and C. Y. contributed equally to this work.

Experimental Section

1. Materials and device fabrication

Tetraethyl orthosilicate (TEOS), bis[3-(triethoxysilyl)propyl]tetrasulfide (BTESPT), γ-chloropropyl trimethoxysilane (CP), 3-aminopropyltriethoxysilane (APTES), etyltrimethylammonium tosylate (CTAT), triethanolamine (TEAH3), triethanolamine (TEA), carboxyl-terminated 50:50 poly(lactic-co-glycolic) acid, fluorescein isothiocyanate (FITC), polyethylenimine linear (Mn 2500), succinic anhydride, carbodiimide hydrochloride (EDC), sulfo-N-hydroxy succunimide (sulfo-NHS), and silica dioxide microparticles with 1 and 2 micron in size were purchased from Sigma-Aldrich Co. (St Louis, MO, USA). Hoechst 33343 bisbenzimide H-33343 trihydrochloride was purchased from VWR. LysoTracker Red DND-99 and Vybrant DiD Cell-Labeling Solution (V22887) were purchased from Thermo Fisher Scientific. ODN 1826-TLR9 ligand and ODN 1926 FITC were purchased from InvivoGen. Infusion/withdrawal PHD ULTRA 4400 pumps were obtained from Harvard Apparatus. All types of MSN (disulfide and diselenide bridged MSN) with different pore size and surface charge were synthesized in our lab as previously described.[1] PLGA NPs were prepared by flash nanoprecipitation method as previously reported using a two-inlets confine impingement jet mixer (CIJ).[2-5] PEI and pDNA encoding GFP protein were dissolved in 10 mM HEPES buffer (pH 7.3) and mixed at volume ratio 1/2 under vortex in a N/P ratio of 6 to prepare GFP-PEI nano-polyplexes. GFP-PEI nano-polyplexes fabricated through FNC were prepared as previously described.[6] CIJ mixer was designed according to literature and fabricated in Columbia University Biomedical Engineering machine shop.[7] A four-stream multi-inlet vortex mixer (MIVM) was manufactured according to literature.[8]

2. CpG loading and releasing from MSN_{NH2}

The optimal CpG loading was achieved with the MSN-to-CpG mass ratio of 5 to 1. A greater of 90% encapsulation efficiency of CpG in MSN_{NH2} was obtained. CpG release from MSNs were evaluated in PBS or 5×10^{-3} M GSH or 1×10^{-4} M H_2O_2 from 0 to 48 h. Briefly, CpG-MSN solution was placed on a shaker with speed of 200 rpm. At each timepoints, the solution was centrifuged and the supernatant was analyzed by UV-Vis.

3. Cell culture

B16-F10 mouse melanoma cells (CRL-6457; American Type Culture Collection), RAW 264.7 mouse macrophage cells (TIB-71l; American Type Culture Collection), HepG2 human liver cancer cells (HB-8065; American Type Culture Collection), Caco-2 human epithelial colorectal cancer cells (HTB-37; American Type Culture Collection), HCT-116 human colon cancer cells (CCL-247; American Type Culture Collection), and HEK 293 human embryonic kidney cells (CRL-1573) were cultured for cell membrane derivation. Cells were cultured in DMEM media with 10% fetal bovine serum (Gibco) and 100 U penicillin-streptomycin.

The generation of bone derived Dendritic cells (BMDCs) followed a previously published protocol.[9] Healthy mice were euthanized using carbon dioxide asphyxiation followed by cervical dislocation. Both femurs were dissected, cleaned in 75% ethanol, and cut on both ends. Bone marrow was then flushed out of the bone with a 1 mL sterile syringe using warm DMEM media containing 10% fetal bovine serum (Gibco) and 100 U penicillin-streptomycin. Cells were then pelleted at 700×g for 5 min, resuspended in BMDC growth media, consisting of the basal media further supplemented with 20 ng/mL granulocyte/macrophage-colony stimulating factor (GM-CSF; Protech), to a concentration of 1×10^{6} cells/mL, and plated into petri plates at 2×10^{6} cells per plate. Media were half-changed every two days.

4. Cell membrane derivation

Cell lines were maintained in DMEM supplemented with 10% FBS and 1% antibiotic/antimycotic solution and incubated at 37 °C with 5% CO_2 in T175 tissue culture flasks. Cells were trypsinized, washed and suspended within PBS. To obtain derived cell membrane, a sequential centrifugation method was applied after lysis and homogenizing of the cells.[1] The resulting membrane pellets were washed and suspended in solution. The total membrane protein contents derived from different cell lines were quantified using BCA protein assay kits separately. Derived cell membrane vesicles were stored in DI water or PBS solution at -80 °C until further use.

5. Cell-membrane-coating and nanoformulation characterization

All particles and cell membrane fragments were prepared and well dispersed in independent solutions. First, the cell membrane vesicles received adequate sonication treatment. Then, particle solutions and cell membrane fragments were introduced into the different inlets of the MIVM respectively. A total of 90-150 mL/min flow rate were applied to prepare membrane-coated particle solution. Raw264.7, B16-F10, Caco-2, HepG2 cell-membranes were used to coat MSNs (small pore) and MSNs (big pore). HEK 293 cell membrane was used to coat silica dioxide microparticles with the membrane/particle mass ratio of 1/2. Caco-2 membrane was used to coat PEI-DNA nanocomplexes with the membrane/particle mass ratio of 1/2. For the surface coating of cationic MSN experiments, both RAW264.7 and B16-F10 membrane were applied with different membrane/MSN mass ratios. The efflux was collected and allowed to settle or stored before further coating characterization. For cell membrane coating NPs through the bulk sonication method, cell membrane vesicles and particle cores at various mass ratios were mixed, pipetted, and sonicated together. B16-F10 membrane was used for all *in vitro* and *in vivo* studies.

Coating efficacy was evaluated based on the membrane protein concentration of the membrane coated product. Membrane coated MSNs by FNC were compared with the

membrane coated bulk-sonication (varied sonication time). Bare MSNs were used as the negative control, and cell membrane vesicles served as the positive control.

The size and zeta potential of naked and membrane-coated particles were examined using a Malvern Zetasizer. The size and zeta potential of MCF membrane-coated PLGA NPs and PEI-DNA polyplexes using bulk-sonication or FNC method were also evaluated using a Malvern Zetasizer. To test the stability of naked and membrane-coated MSNs, particles were stored in solution measured by DLS every other day. For TEM characterization, samples were prepared and dried onto a carbon-coated copper grid with or without uranyl acetate staining before TEM imaging. Identification of gp100 and TRP2 tumor antigen was performed by Western blotting.[9] For gold immunostaining of both FNC based and bulk-sonication based MSN-CpG@CM, nanoparticle solution (100 μL of 20 μg/mL NP) was dropped onto a glow-discharged carbon-coated grid. The grid was then blocked with 5 wt% BSA in 1X PBS and the grid was stained with the anti-gp100 antibody. After washing with the BSA solution, the grid was stained with the gold-conjugated secondary antibody against IgG for 5 minutes. After fixing with 1 wt% glutaraldehyde, the grid was blotted with 20 μL of 0.75% phosphotungstic acid for 1 minute. Imaging was carried out on a Tecnai G2 20TWIN microscope (FEI).

6. Computational fluid dynamics (CFD) and Molecular Dynamics (MD) simulation

The investigation of anionic lipid interaction with the cationic silica nanoparticle was based on the MARTINI force shield while the adopted dissipative particle dynamic (DPD) model was also performed as the small scaled reference for dissipative lipid aggregation analysis.[10–12] Both neural and anionic lipid molecules were used to simulate the lipid composition of cell membrane fragments. The lipid composition was balanced (2000 out of 6600 lipid molecules possess negative charges) and collectively manifested in an anionic manner. The silica NP was constructed using 38882 coarse-grained particles into a nanosphere. Silica (Si-) motif was

partially endowed with positive charge and this rendered the NP with a cationic surface. A cubic box with a PPP boundary and a size of 300 × 300 × 300 nm^3 was created. Then, 6600 lipid molecules are filled into the system in a random position. The interaction between the silica NP and lipid, and theoretical analysis were adopted on the solvent-free MD model developed.[12] The unit command used in the simulation is Lennard-Jones (LJ), and the heat bath used is based on Langevin calculation in LAMMPS. The simulation was run using NVT condition with a temperature of 300 K. Timestep was set to 40 ns per timeframe. All visualization was created using the dump command and run on the software Ovito Pro 3.0. The commercial CFD solver Ansys FLUENT ver. 19.2 was used to approximately solve the Navier-Stokes equations. A turbulence model of κ-ε was adopted to capture the gradually varying flow from the inner region to the interior lumen. The turbulence intensity was set to 1. A hybrid mesh consisting of tetrahedral grids inside and a prismatic boundary layer was generated using the ANSYS meshing system in ANSYS workbench, which contained 166416 cells and 393820 grids with an inflation layer with eight layers and a growth rate of 1.1. The liquid used in the simulation was water of a density ρ = 1000 kg·m^{-3}, of a dynamic viscosity μ=0.8904×10^{-3} Pa·s. The shear stress transport eddy-viscosity model was adopted to capture the WSS distribution. We set the spatial discretization as second-order upwind, and the truncation error was second-order. The lumen wall was set to be rigid with a no-slip boundary condition. A boundary condition strategy was designed with four inflow inlets and a pressure outlet in the mixer. In this study, we used a steady inflow boundary condition of mass flow rates of 0.0005, 0.0015, 0.0025 kg/s and 0 pressure outflow condition.

7. Cytotoxicity assay

The cytotoxicity of MSN, MSN-CpG and MSN-CpG@CMs in the RAW264.7 or BMDC were assessed using an MTT assay. The assay was performed in a 96-well plate containing 5×10^3 cells per well. The cells were cultured in complete medium containing different

concentrations (0, 12.5, 25, 50, 100 and 200 μg/mL) of substances for 24 h, and an MTT assay was performed. The optical density of each well was measured by a multifunctional microplate reader at a wavelength of 490 nm. The relative survival rate (mean (%) ± SD, n=6) of the cells was calculated using the following equation: survival rate (%) = ($A490_{\text{treated sample}}$ / $A490_{\text{untreated sample}}$) × 100%.

8. ***In vitro*** **uptake and activity**

For the cellular uptake study, BMDCs were collected on day 5 and plated into 24-well suspension plates. FAM-labeled CpG, MSN-CpG and MSN-CpG@CMs were added at an equivalent CpG concentration of 5 μg/mL. After 3 h incubation, the cells were washed and stained with DAPI and LysoTracker Red. 15 min later, cells were imaged by using a laser scanning confocal microscopy (CLSM). For flow cytometry, cells were collected, washed twice in PBS, and resuspended in 200 μL of 10% PBS. The cell suspension was analyzed using BD Accuri C6 plus flow cytometer. Collected data were analyzed by FlowJo software. The activity of the delivered CpG was examined using a BMDC maturation assay and cytokine release assay. BMDCs were collected on day 5, and 3×10^6 BMDCs were plated into 6-well suspension plates in BMDC growth media. Cells were pulsed with materials for 12 h at 5 μg/mL CpG, then washed twice with fresh media. After an additional 48 h of culture, cell supernatants were collected and cytokine content was analyzed using IL-6 and IL-12 ELISA kits. The cells were then collected, washed twice and stained with FITC-conjugated anti-mouse CD11c and APC-conjugated anti-mouse CD40, CD80 or CD86. Appropriate dye-labeled antibody isotypes (Biolegend) were used for gating purposes with cells from an untreated lymph node. Data were collected using a BD FACSCelesta flow cytometer and analyzed using FlowJo software. RAW264.7 cells were plated into 6-well suspension plates at 5×10^5 cells/well and pulsed with materials for 24 h at 5 μg/mL CpG, then cell supernatants were collected and cytokine content was analyzed using TNF-α ELISA kits.

9. *In vivo* lymph node distribution and DC activation

All animals received care in compliance with the guidelines outlined in the Guide for the Care and Use of Laboratory Animals, and the procedures were approved by the South China University of Technology Animal Care and Use Committee. Female C57BL/6J mice were obtained at 6–10 weeks old from Hunan SJA Laboratory Animal Co., LTD.

Materials containing Cy5.5-labeled CpG or DiD-labeled membrane fragments were used to trace the distribution of nanovaccines in lymph nodes. After injecting different materials at foot pad for 1, 3, 6, 12, 24, and 48 h, female C57BL/6J mice were euthanized and their popliteal lymph nodes were collected. Dye-labeled nanovaccines, 20 μL at 1 mg/mL, were injected into both hocks of female C57BL/6NJ mice. At time points of 6, 12, 24, and 48 h, the popliteal lymph nodes were collected. All the lymph nodes were analyzed and quantified by In-Vivo Xtreme Imaging System (Bruker).

To further assess the cellular uptake in lymph node, dye-labeled nanovaccines were injected subcutaneously into each hock of female C57BL/6J mice. After 24 h, the popliteal lymph nodes were collected, dissociated manually by pipetting, then stained with antibodies for dendritic cells (CD11c monoclonal antibody, 12-0114-82; eBioscience) and macrophages (F4/80 monoclonal antibody, 11-4801-82; eBioscience) for 30 min. Data were collected using BD FACSCelesta flow cytometer and analyzed using FlowJo software.

Dendritic cell activation following immunization with CpG, MSN-CpG, and MSN-CpG@CMs was determined by testing DC maturation and lymph node cytokine secretion. To examine DC maturation *in vivo*, 20 µL of each material was injected into the hock. After 24 h, the popliteal lymph nodes of all treated mice were collected into 500 µL dissociation buffer and manually dissociated. Cells were stained using PE antimouse CD11c with either APC-conjugated antimouse CD40 (124611; Biolegend), CD80 (104713; Biolegend), or CD86 (105011; Biolegend). Data were collected using a Becton Dickinson FACSCanto-II FLow

cytometer and analyzed using FlowJo software. To analyze cytokine production, lymph node-derived single-cell suspensions were plated with 500 µL of BMDC growth media in 24-well tissue culture plates. After 48 h, supernatant was collected and analyzed for cytokine content using IL-6 and IL-12 ELISA kits.

To assess the generation of antigen-specific T cells, C57BL/6J mice were vaccinated subcutaneously with 20 µL of the different materials on days 0, 2, and 4. On day 10, spleens were collected and processed into single cell suspensions. After lysis of red blood cells, 5×10^6 splenocytes were plated onto 6-well suspension plates and pulsed with 1 µg/mL of mouse gp100 peptide with sequence EGSRNQDWL in BMDC growth media. After 7 days, cells were collected, washed in PBS solution, and stained with APC-conjugated anti-mouse CD8a and phycoerythrin (PE)-labeled H-2Db gp100 tetramer. Data were collected using a BD FACSCelesta flow cytometer and analyzed using FlowJo software. To evaluate the immunological effects of the combined treatment, B16-F10 tumor-bearing mice were randomly divided into six groups (n=3 mice in each group). When the tumor volume reached approximately ~100 mm^3, the mice were treated with different materials at day 1 and 4. After 7 days of treatment, the mice were sacrificed, and the tumors were collected for immunological evaluations. To evaluate the immune cells in tumors, tumors collected from different groups of mice were cut into small pieces and homogenized in PBS (pH 7.4) containing 0.5% FBS to obtain suspensions of single cells, which were then stained with corresponding antibodies after the removal of red blood cells (RBC) using RBC lysis buffer (Solarbio). To identify CD4+cells (CD3+CD4+CD8-), CD8+cells (CD3+CD4-CD8+) and regulatory T cells (Treg cells) (CD3+CD4+Foxp3+), the following antibodies (from eBioscience, Biolegend) were used, respectively: CD4 monoclonal antibody (GK1.5, Alexa Fluor 488), CD8a monoclonal antibody (53-6.7, PE), CD3e monoclonal antibody (145-2C11, APC), True-Nuclear™ Mouse Treg Flow™ Kit (FOXP3 Alexa Fluor® 488/CD4 APC/CD25 PE), and TruStain FcX™ (anti-mouse CD16/32).

10. *In vivo* anticancer immunity and immunotherapeutic effects

To study the tumor prevention effect conferred by vaccination, C57BL/6J mice were vaccinated with 100 µL of the different materials at 0.1 mg/mL of CpG or equivalent, on days -21, -14, and -7. On day -1, the right flank of each mouse was shaved and, on day 0, mice (n=6) were challenged with 2×10^4 B16-F10 cells subcutaneously on the right flank. The tumor size was measured every other day and the experimental endpoint was defined as either death or tumor size greater than 2000 mm^2.

To study the therapeutic effect, C57BL/6J mice were first challenged on the right flank with 1×10^5 B16-F10 cells on day 0. On day 2, 4, and 7, mice (n=6) were vaccinated subcutaneously in the same flank with 100 µL of the materials. The checkpoint blockade cocktail, consisting of 100 µg anti-CTLA4 (BP0164; BioXCell) was administered intraperitoneally on the same days. Tumors were measured every other day and the experimental endpoint was defined as either death or tumor size greater than 2000 mm^2.

11. Statistical analysis

Data were expressed as mean ± SD. Differences between groups were analyzed using Student's t-test when comparing only two groups. Differences among more than two groups were analyzed using one-way analysis of variance, and the Bonferroni post hoc test was used to analyze the differences between any two groups. $P < 0.05$ was considered representative of a statistically significant difference. GraphPad Prism 8 was used to perform the statistical analysis.

References

[1] D. Shao, M. Li, Z. Wang, X. Zheng, Y.-H. Lao, Z. Chang, F. Zhang, M. Lu, J. Yue, H. Hu, H. Yan, L. Chen, W. Dong, K. W. Leong, *Adv. Mater.* **2018**, *30*, 1801198.
[2] G. P. Howard, G. Verma, X. Ke, W. M. Thayer, T. Hamerly, V. K. Baxter, J. E. Lee, R. R. Dinglasan, H.-Q. Mao, *Nano Res.* **2019**, *12*, 837.
[3] W. S. Saad, R. K. Prud'homme, *Nano Today* **2016**, *11*, 212.
[4] Z. Zhu, *Biomaterials* **2013**, *34*, 10238.
[5] H. Shen, A. A. Banerjee, P. Mlynarska, M. Hautman, S. Hong, I. M. Kapetanovic, A. V. Lyubimov, Y. Liu, *J. Pharm. Sci.* **2012**, *101*, 3877.
[6] Z. He, Y. Hu, T. Nie, H. Tang, J. Zhu, K. Chen, L. Liu, K. W. Leong, Y. Chen, H.-Q. Mao, *Acta Biomater.* **2018**, *81*, 195.
[7] B. K. Johnson, R. K. Prud'homme, *AIChE J.* **2003**, *49*, 2264.
[8] Z. He, J. L. Santos, H. Tian, H. Huang, Y. Hu, L. Liu, K. W. Leong, Y. Chen, H.-Q. Mao, *Biomaterials* **2017**, *130*, 28.
[9] A. V. Kroll, R. H. Fang, Y. Jiang, J. Zhou, X. Wei, C. L. Yu, J. Gao, B. T. Luk, D. Dehaini, W. Gao, L. Zhang, *Adv. Mater.* **2017**, *29*, 1703969.
[10] R. D. Groot, P. B. Warren, *J. Chem. Phys.* **1997**, *107*, 4423.
[11] L. Zhang, Q. Feng, J. Wang, J. Sun, X. Shi, X. Jiang, *Angew. Chem. Int. Ed.* **2015**, *54*, 3952.
[12] J. Sun, L. Zhang, J. Wang, Q. Feng, D. Liu, Q. Yin, D. Xu, Y. Wei, B. Ding, X. Shi, X. Jiang, *Adv. Mater.* **2015**, *27*, 1402.

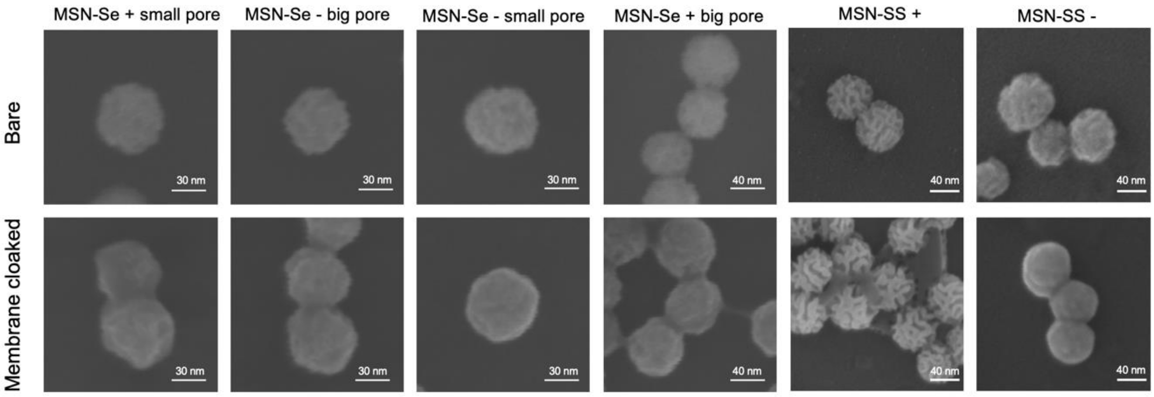

Figure S1. SEM images of bare cores and cell membrane-coated particles produced using FNC.

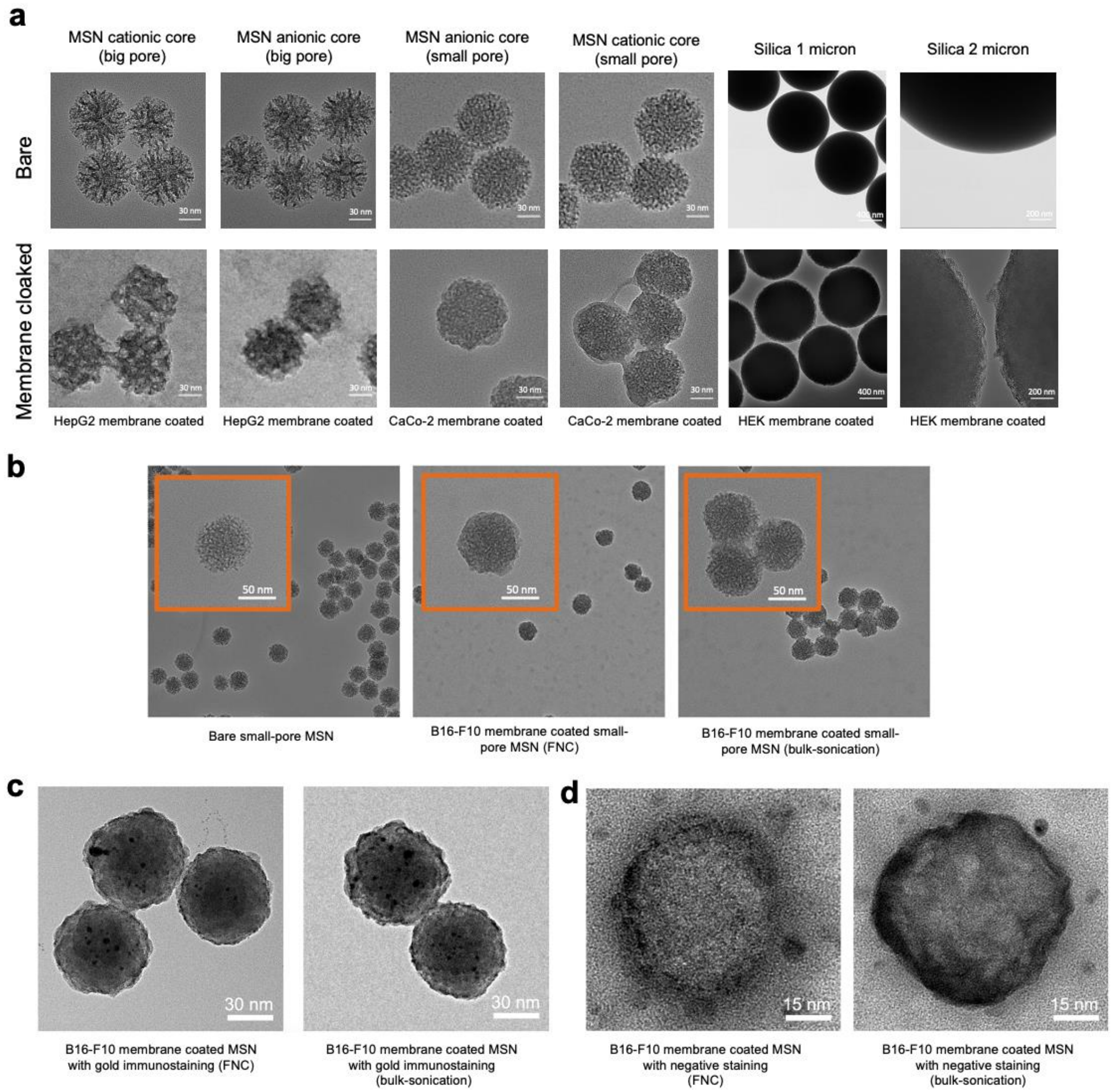

Figure S2. TEM images of a) bare cores and cell membrane-coated particles produced using FNC. b) uncoated MSN small pore, B16-F10 melanoma cell membrane-coated small-pore MSN by FNC method, and by the bulk-sonication method, respectively. c) B16-F10 melanoma cell membrane-coated MSN by FNC method and bulk-sonication method with anti-gp100 gold immunostaining, respectively, and d) B16 melanoma cell membrane-coated MSN by FNC method and bulk-sonication method with negative staining, respectively.

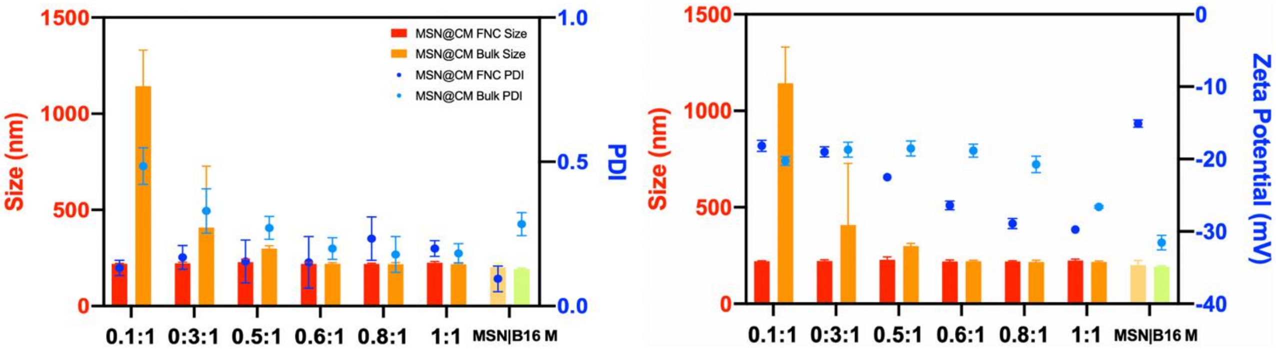

Figure S3. Size, PDI, and Zeta potential of B16-F10 membrane-coated anionic MSN-Se NPs produced using bulk sonication or FNC methods. Data represent mean ± SD (n=3).

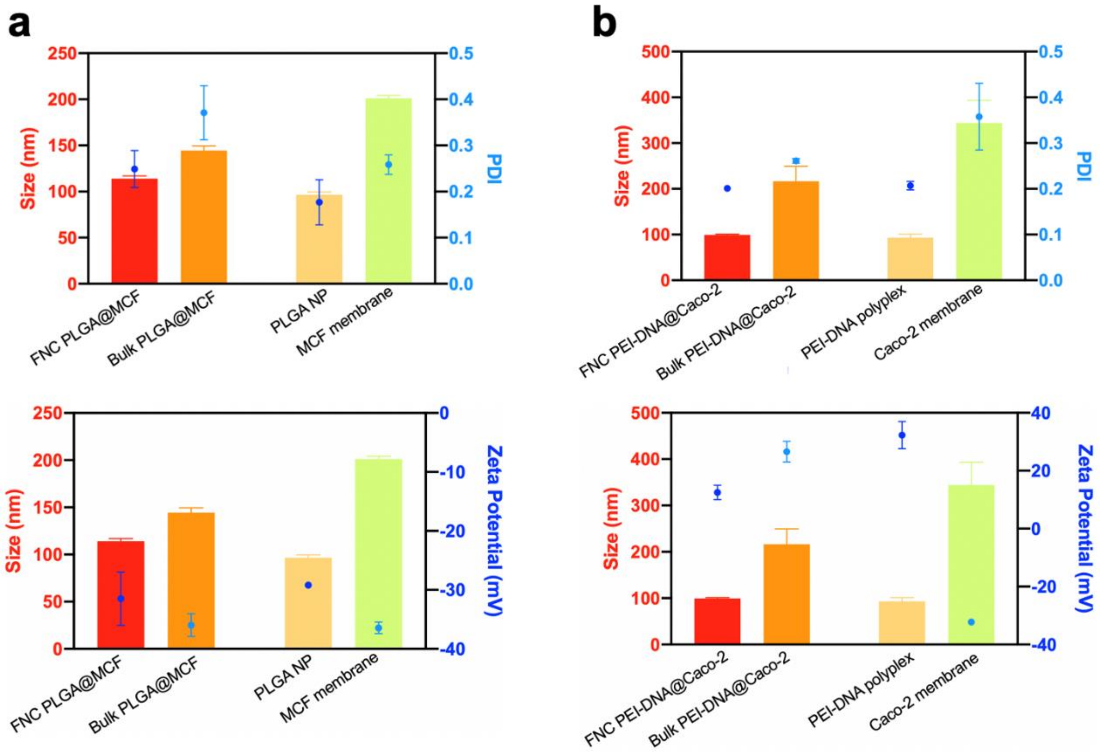

Figure S4. Size, PDI, and Zeta potential of a) MCF membrane-coated PLGA NPs, and b) Caco-2 membrane-coated PEI-plasmid NPs, produced by bulk sonication or FNC methods. Data represent mean ± SD (n=3).

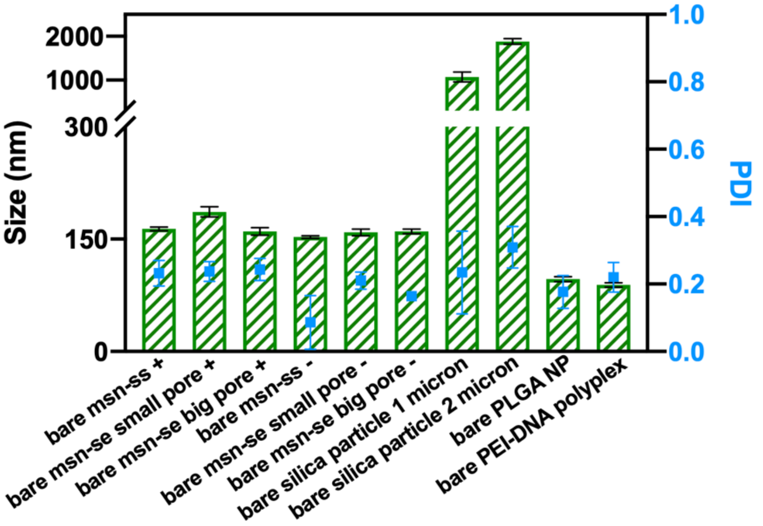

Figure S5. DLS characterized size and PDI of different bare particle cores prepared for cell-membrane coating. Data represent mean ± SD (n=3).

Particle name	Particle abbreviation	Bare size (in solution)	Cell membrane involved
Cationic diselenide MSN*	MSN-Se_{NH2} or MSN-Se +	~ 150 nm	RAW264.7 & B16-F10 & HepG2 & Caco-2
Anionic diselenide MSN*	MSN-Se_{COOH} or MSN-Se -	~ 150 nm	RAW264.7 & B16-F10 & HepG2 & Caco-2
Cationic disulfide MSN	MSN-SS_{NH2} or MSN-SS +	~ 150 nm	HepG2
Anionic disulfide MSN	MSN-SS_{COOH} or MSN-SS -	~ 150 nm	HepG2
SiO_2 particle with 1 μm diameter size	Silica particle 1 micron	~ 1 μm	HEK 293
SiO_2 particle with 2 μm diameter size	Silica particle 2 micron	~ 2 μm	HEK 293
PLGA nanoparticle	PLGA NP	~ 100 nm	MCF-7
PEI-DNA (GFP) polyplex	PEI-DNA polyplex	~ 90 nm	Caco-2
CpG-loaded cationic diselenide MSN	MSN-CpG	~ 180 nm	-
Cell membrane coated CpG-loaded cationic diselenide MSN	MSN-CpG@CM	~190 nm	B16-F10

*diselenide MSNs were synthesized with big pore and small pore.

Table S1. Summary of particle names, particle abbreviations, size and cell-membrane types used in the coating experiments.

Sonication (n = 9)	FNC (n = 3)	Mixing – Pipette (n = 3)
29.04% ± 11.85 (30 secs) (n = 3)	59.65% ± 5.20	12.76% ± 9.59
44.16% ± 31.90 (2 mins) (n = 3)		
38.07% ± 11.25 (10 mins) (n = 3)		

Table S2. Coating efficacy of the cell membrane-coated anionic MSN in bulk and FNC method.

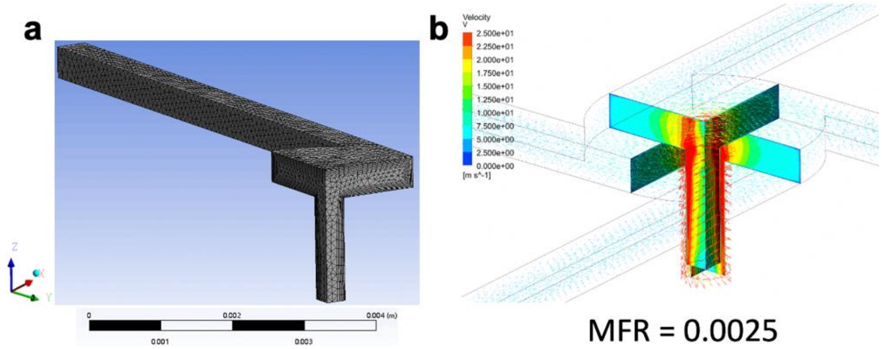

Figure S6. CFD simulation. a) Construction of a quarter of MIVM model for shear and velocity analysis in the CFD. b) Analysis of the mass flow rate at 0.0025 kg/s within the MIVM.

a

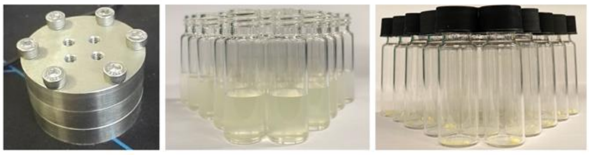

b

MIVM flow rate (mL/min)	Production yield (g/h)	
	C_{MSN} = 1 mg/mL	C_{MSN} = 5 mg/mL
60	1.8	9.0
120	3.6	18.0
166	5.0	25.0

Figure S7. Fabrication of cell membrane-coated nanoparticles using flash nanocomplexation. a) Images of multi-inlet vortex mixer (MIVM) and vials containing total of 40 mL B16-F10 membrane-coated MSNs at 0.5 mg/mL as well as their lyophilized product. b) The production yield for biomimetic MSN nanoformulation using FNC at different flow rates.

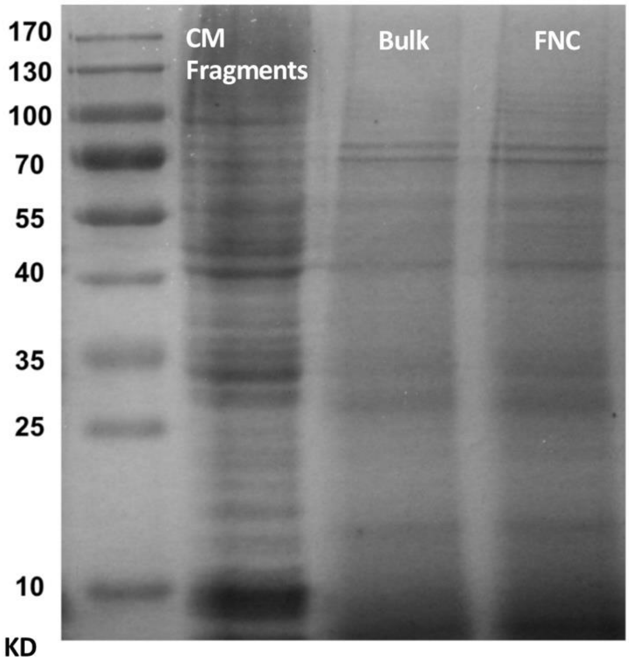

Figure S8. SDS-PAGE protein analysis of MSN-CpG@CM produced using bulk sonication or FNC methods.

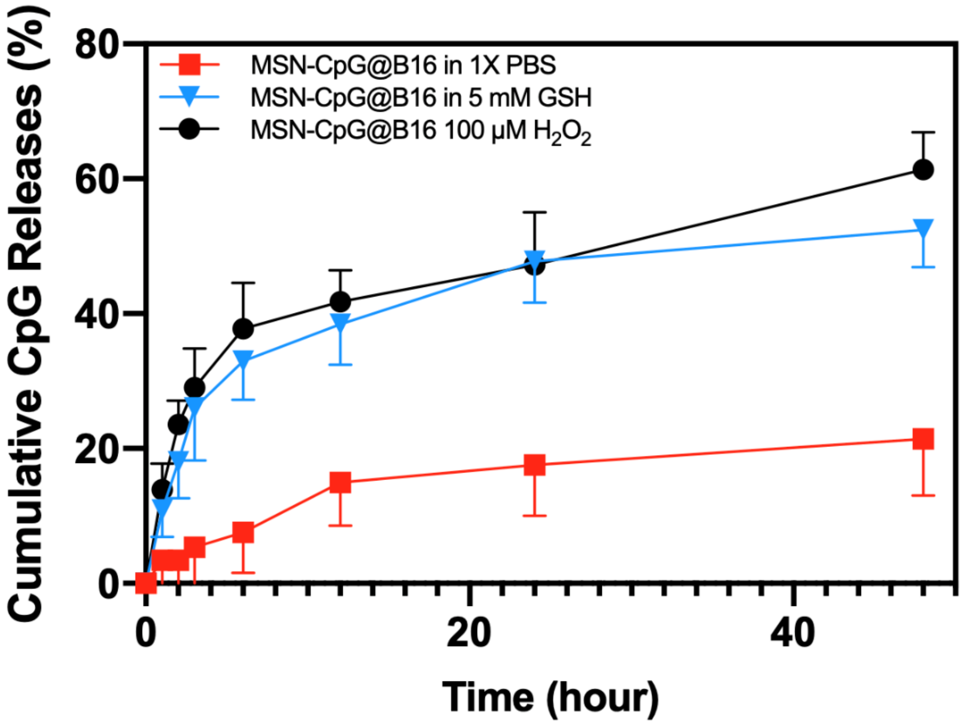

Figure S9. CpG release behavior of MSN-CpG@CM produced using FNC in 1 × PBS or 5×10^{-3} M GSH or 1×10^{-4} M H_2O_2 for 48 h.

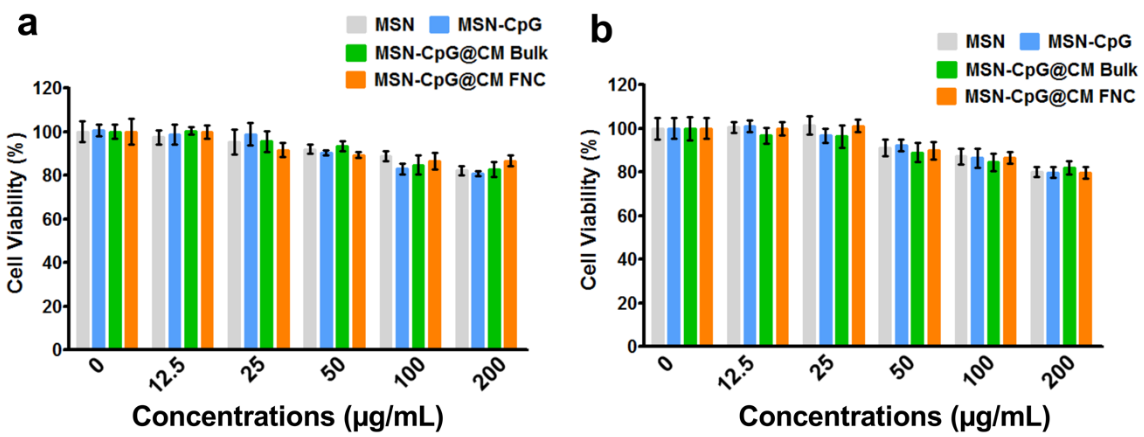

Figure S10. Cell viability of a) BMDCs, and b) RAW264.7 cells incubated with various concentrations of MSN, MSN-CpG, or MSN-CpG@CM produced by bulk sonication or FNC methods for 24 h. Data represent mean ± SD (n=3).

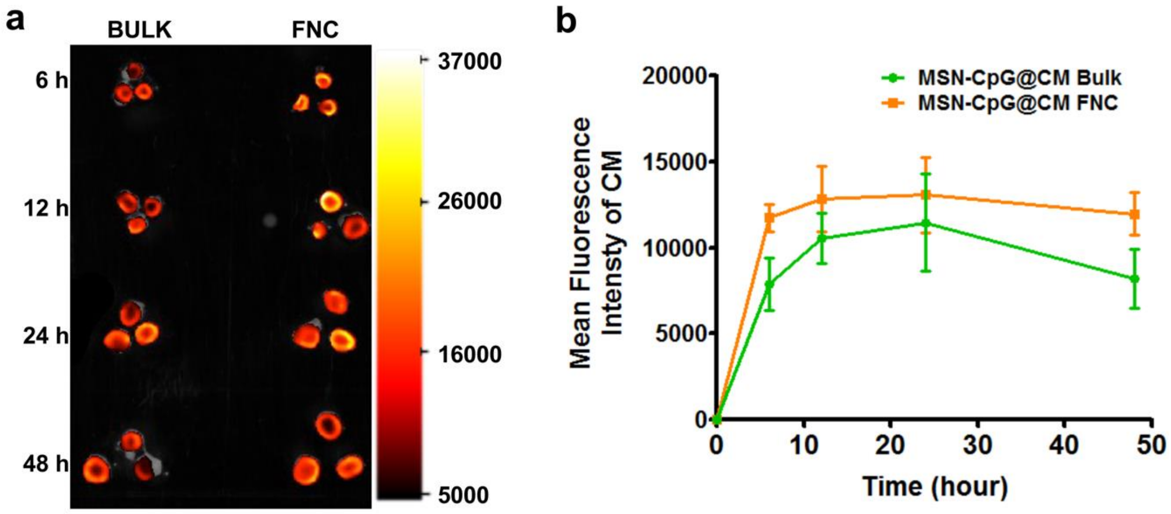

Figure S11. a) Fluorescence imaging of popliteal lymph node at indicated time points after footpad injection of MSN-CpG@CM produced using bulk sonication or FNC methods. b) Quantitative fluorescence intensity of DiD-labeled membranes in lymph node. Data represent mean ± SD (n=3).

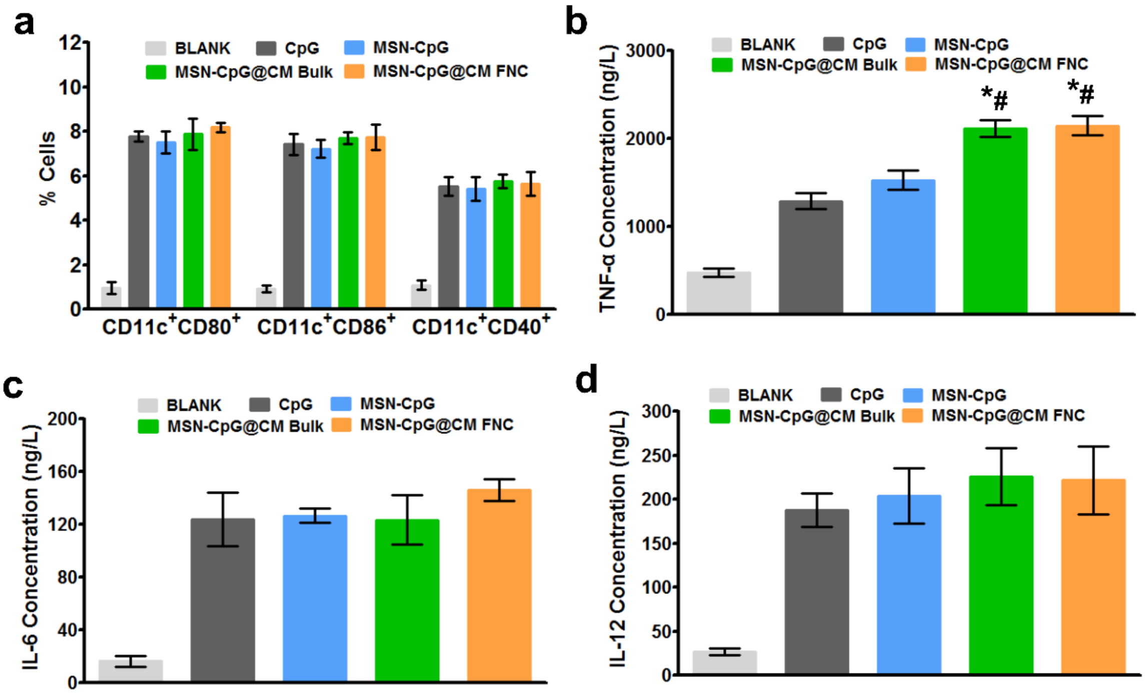

Figure S12. APCs were incubated with nanovaccines or various control formulations. a) Quantification of DC maturation markers CD40, CD80, CD86 *in vitro*. b) Secretion of TNF-α in macrophage (RAW 264.7) suspensions measured by ELISA. Secretion of c) IL-6, and d) IL-12 in DC suspensions measured by ELISA. Data represent mean ± SD (n=3, $^{*}p < 0.05$ vs. CpG group, $^{\#}p < 0.05$ vs. MSN-CpG group).

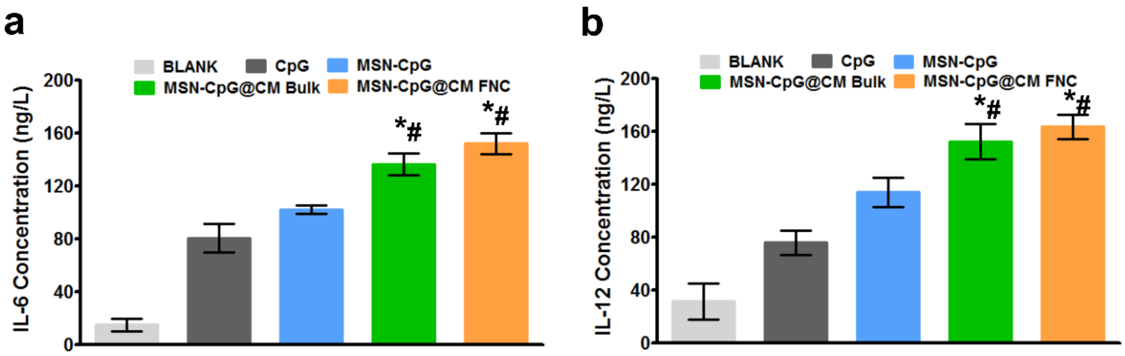

Figure S13. Secretion of a) IL-6 and b) IL-12 in DCs isolated from popliteal lymph nodes after vaccination with nanovaccines or control formulations. Data represent mean ± SD (n=3, $^{*}p < 0.05$ vs. CpG group, $^{\#}p < 0.05$ vs. MSN-CpG group).

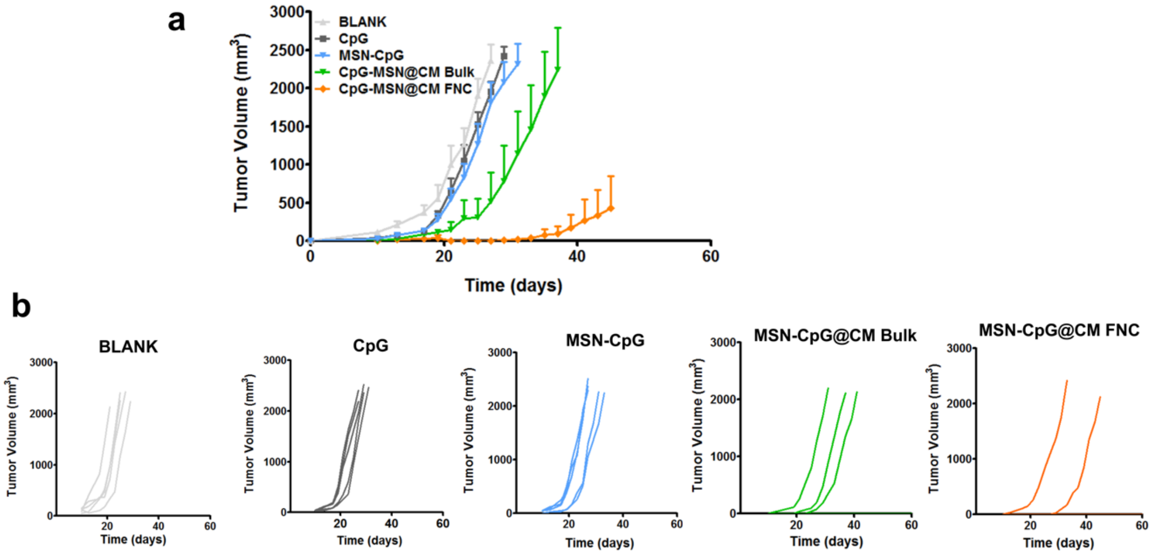

Figure S14. a) Average tumor sizes and b) individual tumor growth kinetics for nanovaccines in prophylactic melanoma model (n=6).

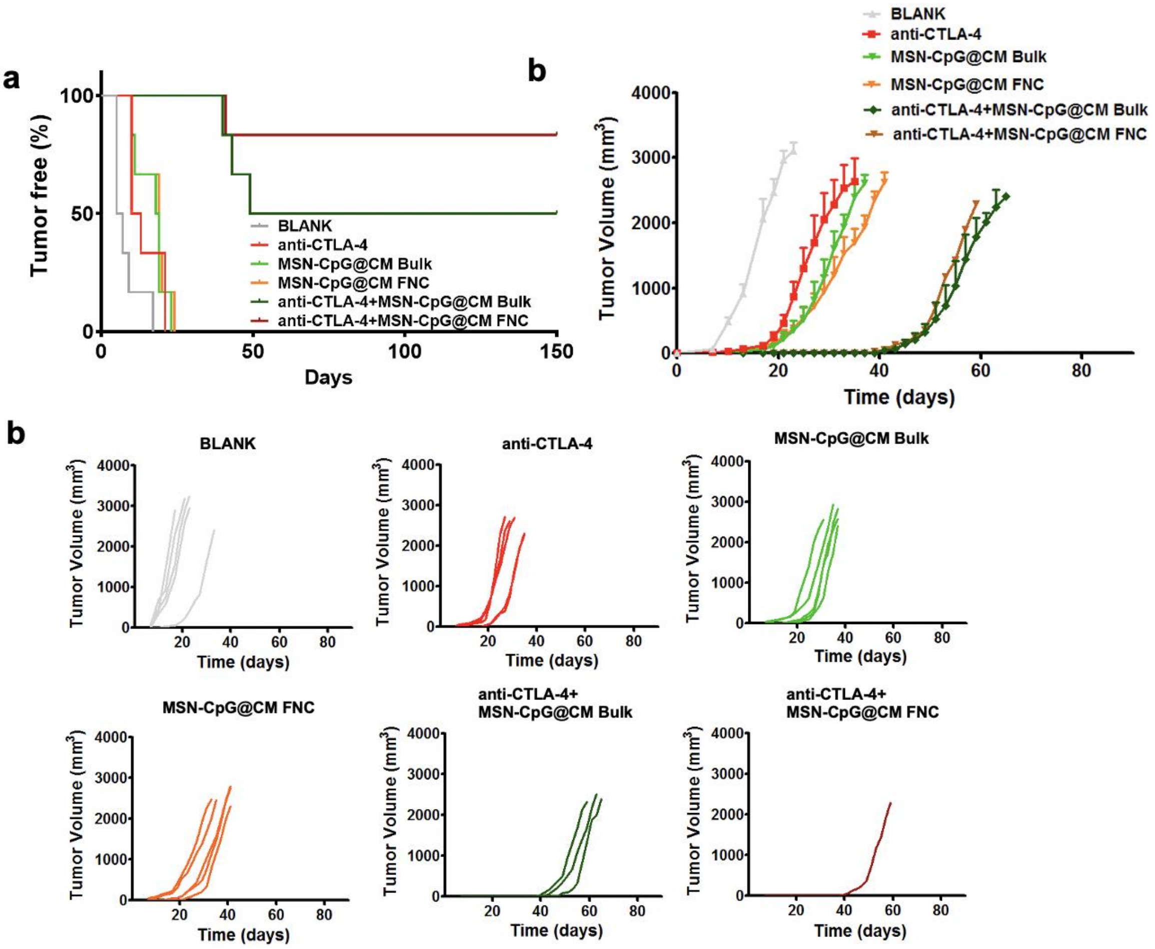

Figure S15. a) Tumor free percentage, b) average tumor sizes, and c) individual tumor growth kinetics for nanovaccines with or without the checkpoint blockade inhibitor anti-CTLA-4 in melanoma model (n=6).

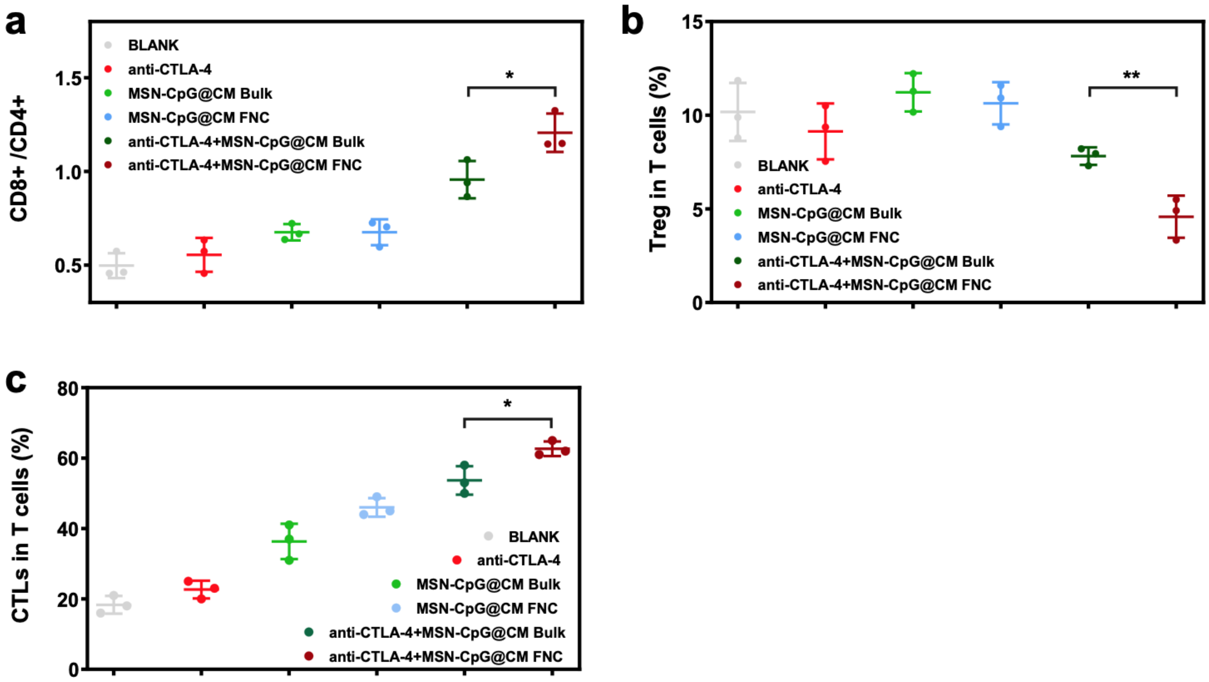

Figure S16. Relative composition of T cells within the tumor in the melanoma model after administration of nanoformulations with or without the checkpoint blockade inhibitor anti-CTLA-4 (n=3). a) Ratio of CD8+ to CD4+ T cells, b) percentage of regulatory T cells, c) percentage of cytotoxic T lymphocytes.
